# Supplementary material for: Real-space imaging with pattern recognition of a ligand-protected Ag374 nanocluster at sub-molecular resolution
Source: Nat Commun. 2018 Jul 27;9:2948. doi: 10.1038/s41467-018-05372-5 (PMC6063937; doi:10.1038/s41467-018-05372-5)
Supplement: Supplementary file 1 — Supplementary Information [file 41467_2018_5372_MOESM1_ESM.pdf]

SUPPLEMENTARY INFORMATION FOR

Real-Space Imaging with Pattern Recognition of a Ligand-Protected Ag_{374} Nanocluster at Sub-Molecular Resolution

Zhou et al.

SUPPLEMENTARY FIGURES

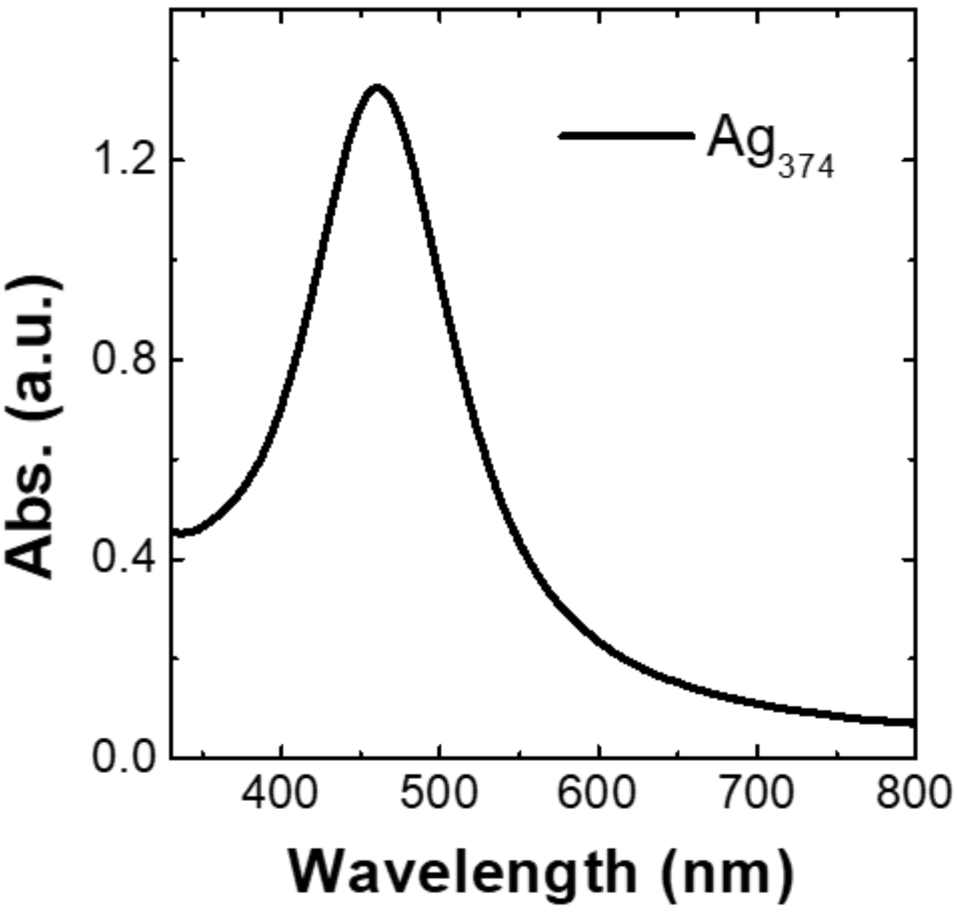

Supplementary Figure 1. Optical absorption of Ag_{374} as dissolved from crystalline phase before depositing on the dithiol-modified gold surface.

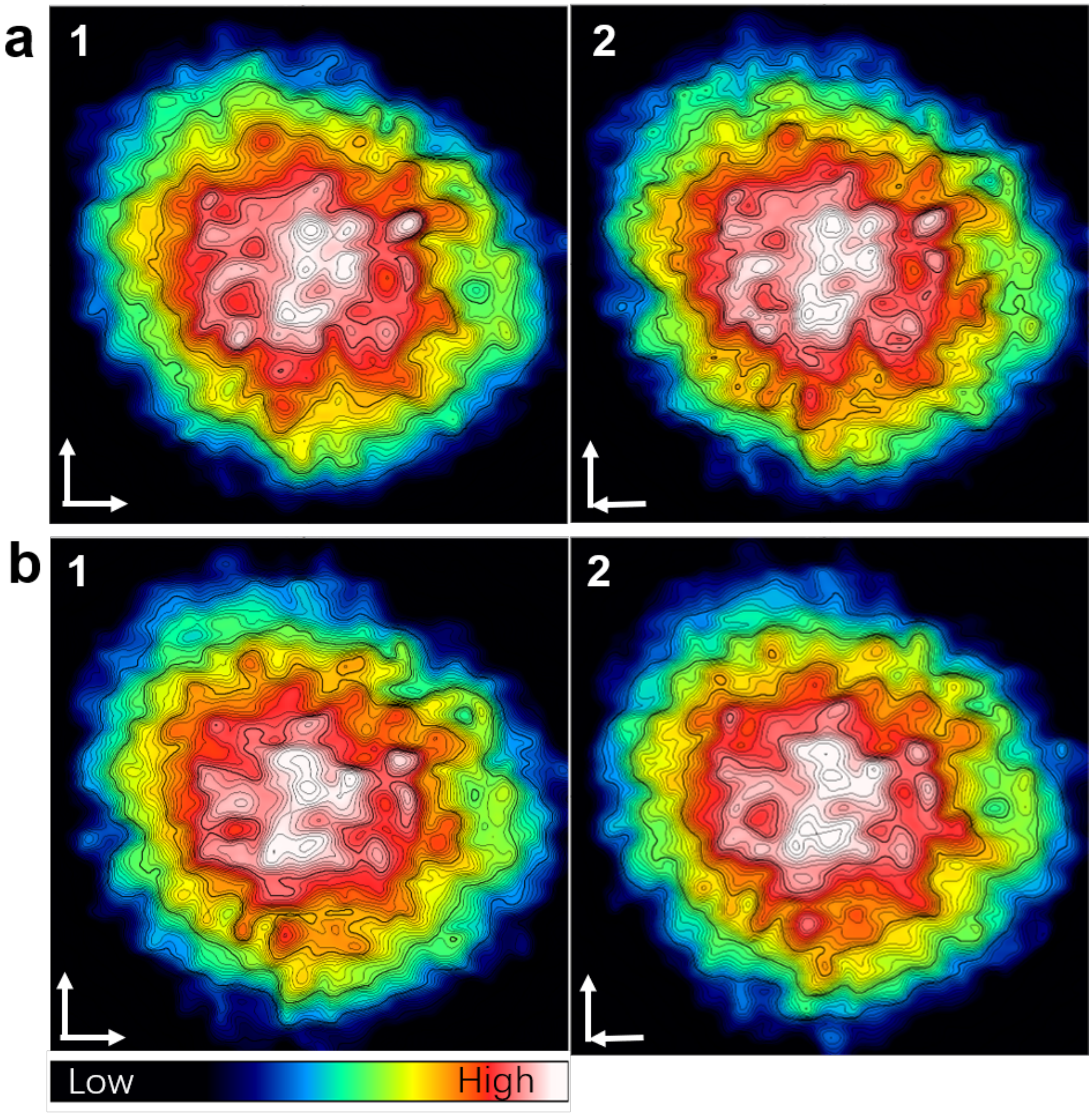

Supplementary Figure 2. Repeated scans of the same Ag_{374} cluster at LHe temperature. Scan directions are shown by the arrows. **(1)** and **(2)** in (**a,b**) refer to up (or down) images with opposite scanning directions (trace and retrace) marking at left bottom which were recorded simultaneously at a time scale of 7min. Bias voltage: -1.2V, 10pA. 5.0 $\times$ 5.0 nm. Images in **(b)** （**1,2**） were selected in calculation in Fig. 4 as the references.

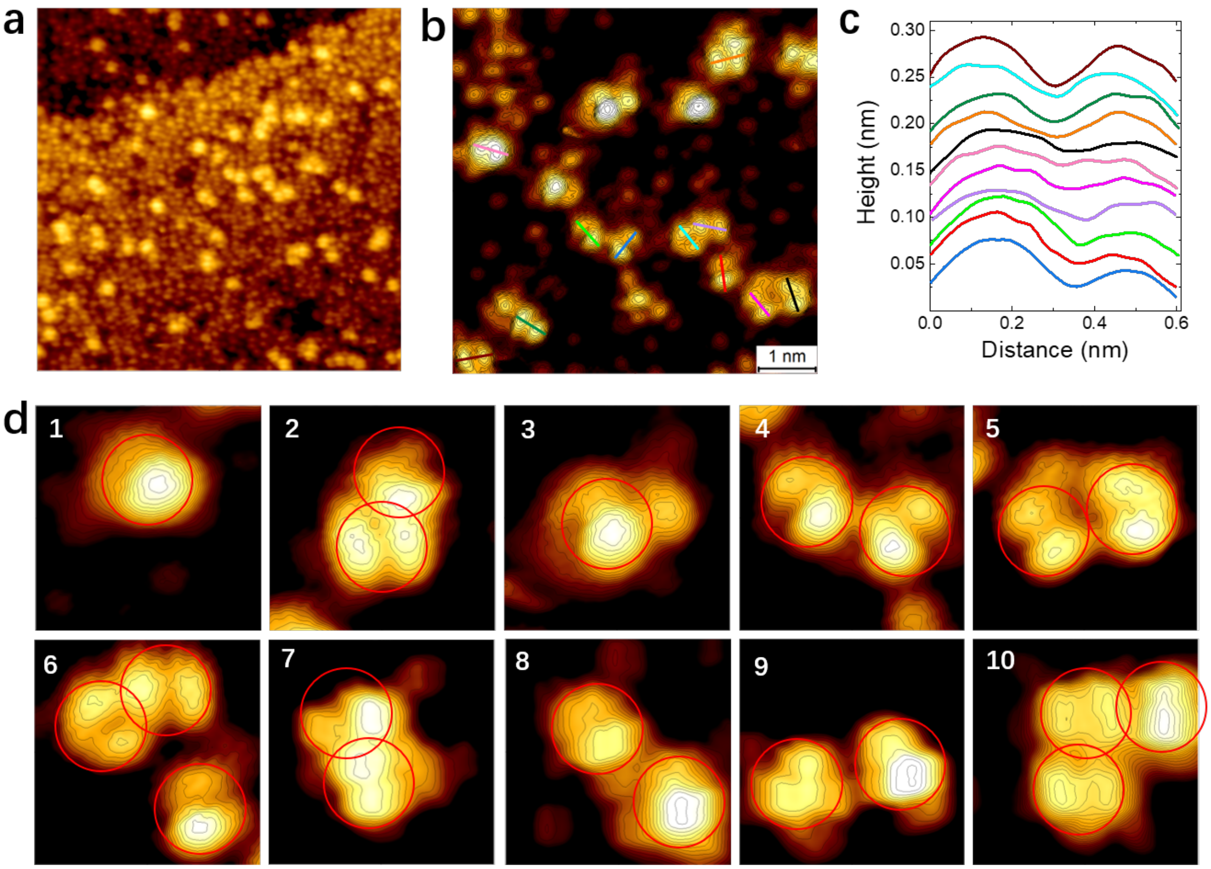

Supplementary Figure 3. STM images of the ligand database. (**a**) large scale image where TBBT molecules adsorbed on Au(111) surface with multilayers. Size: 21.5 x 21.5 nm^2, bias and setpoint current: 1.2 V, 40 pA. (**b**) Zoom-in image with bright individual TBBT molecules on the outmost surface. Size: 6.38 ×6.38 nm^2, bias and setpoint current: 1.2 V, 60 pA. (**c**) Height profile for colored lines marked in (**b**), which were shifted for good visual. (**d**) Part of TBBT image database, each red circle (0.6 nm diameter) indicates one TBBT molecule. All the images were digitally zoomed-in from large area images. Size, bias and setpoint current**: (1-6)** 1.51 × 1.51 nm, 1.2V, 60pA, **(7-10)** 1.50 × 1.50 nm, 1.0V, 30pA. Measured at LN_2 temperature.

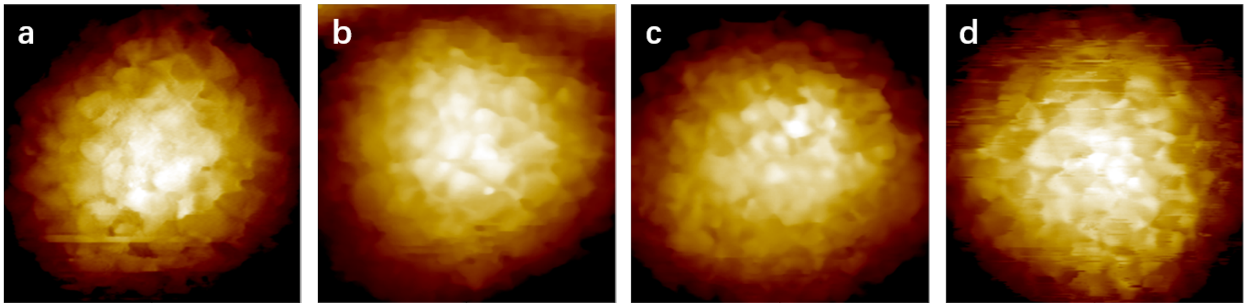

Supplementary Figure 4. High-resolution images of Ag_{374} at LN_2 temperature. Four different clusters showing a similar degree of high sub-molecular resolution as shown in Fig. 3 of the main text at LHe temperature. Bias, setpoint current, scanning angle, size: **(a)** -1.4 V, 20 pA, 0°, 4.81 × 4.81 nm^2; **(b)** -1.5 V, 40 pA, 0°, 4.81 × 4.81 nm^2 ; **(c)** -1.5 V, 30 pA, 90°, 4.80 × 4.80 nm^2 ; (**d)** -1.2 V, 30 pA, 45°, 4.80 × 4.80 nm^2.

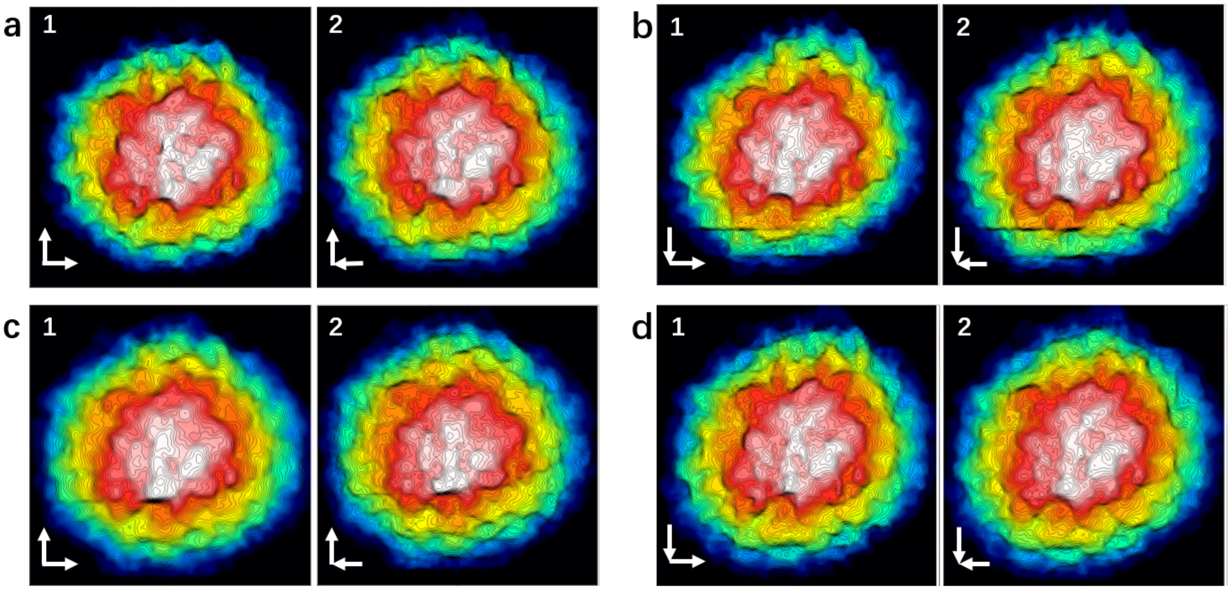

Supplementary Figure 5. Continuous scanning of the same Ag_{374} cluster at LN_2 temperature. Scan directions are shown by the arrows. **(1)** and **(2)** in (**a-d**) refer to up (or down) images with opposite scanning directions (trace and retrace) marking at left bottom which were recorded simultaneously at a timescale of 8 min 45 sec. Size: 4.81 × 4.81 nm^2. Bias and setpoint current: -1.4 V, 20 pA.

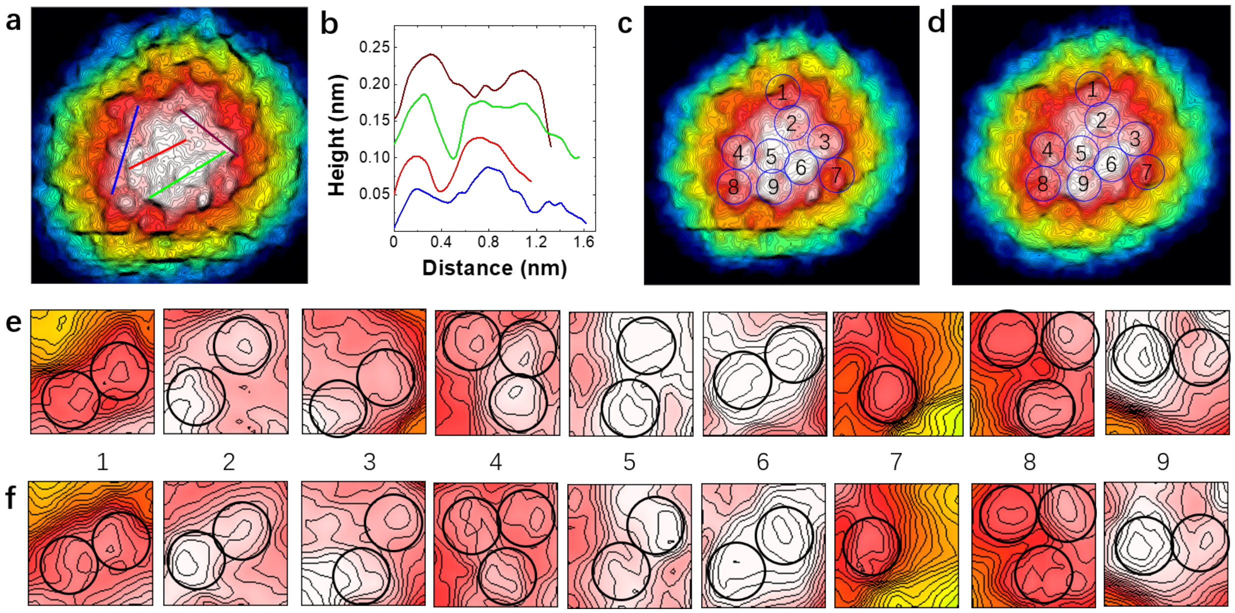

Supplementary Figure 6. High resolution image of Ag_{374} and the detailed ligand information. **(a)** the same as Fig. S5 b2. **(c)** and **(d)** same as Fig. S5 b2 and d2, respectively. **(b)** Height profile of colored lines marked in **(a).** The numbered circles 1-9 (diameter 0.6 nm) refer to local areas shown zoomed-in in **(e,f).** Size: 4.81 × 4.81 nm^2. Bias and setpoint current: -1.4 V, 20 pA. **(e,f)** blow-up of areas 1-9 from **(c)** and **(d),** respectively. Black circles denote local maxima.

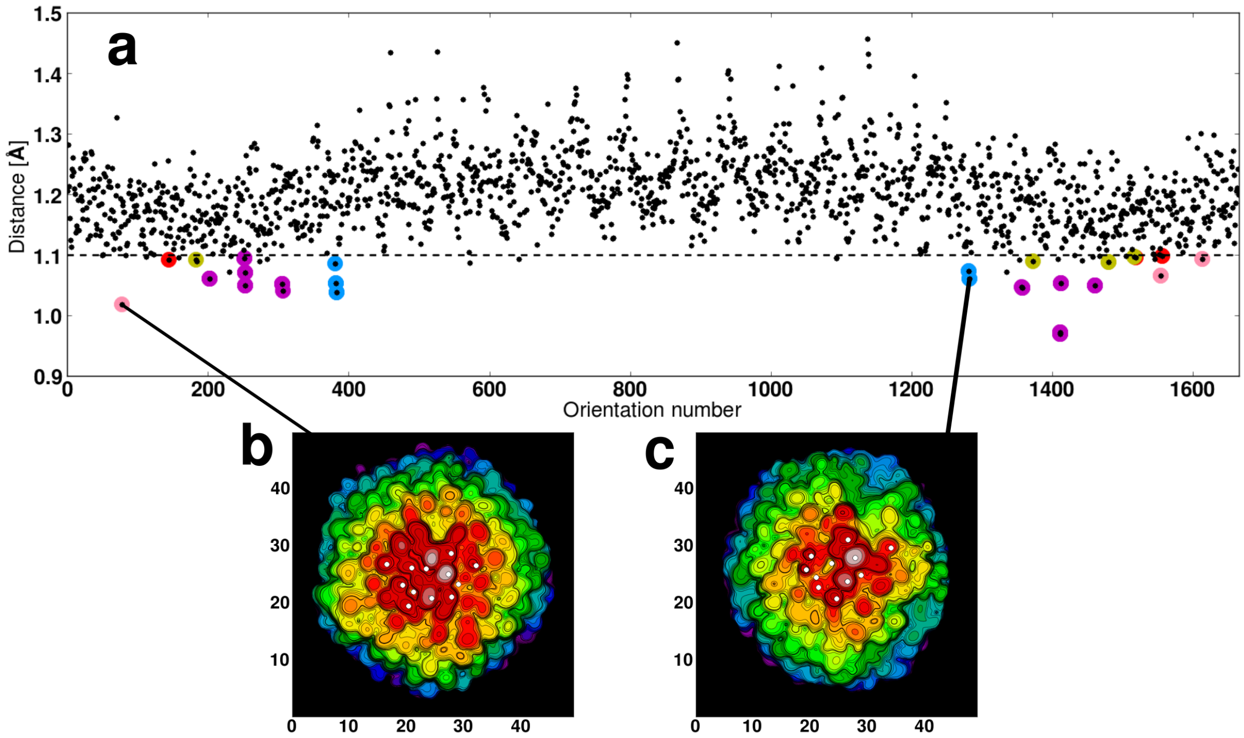

Supplementary Figure 7. The facial recognition of the retrace STM data (Fig. 4b). (**a**) Distance analysis for the STM image in figure 4b. Selected small-distance perspectives are connected with lines to the respective calculated STM images (**b**,**c**). The colors are the same as in figure 4d.

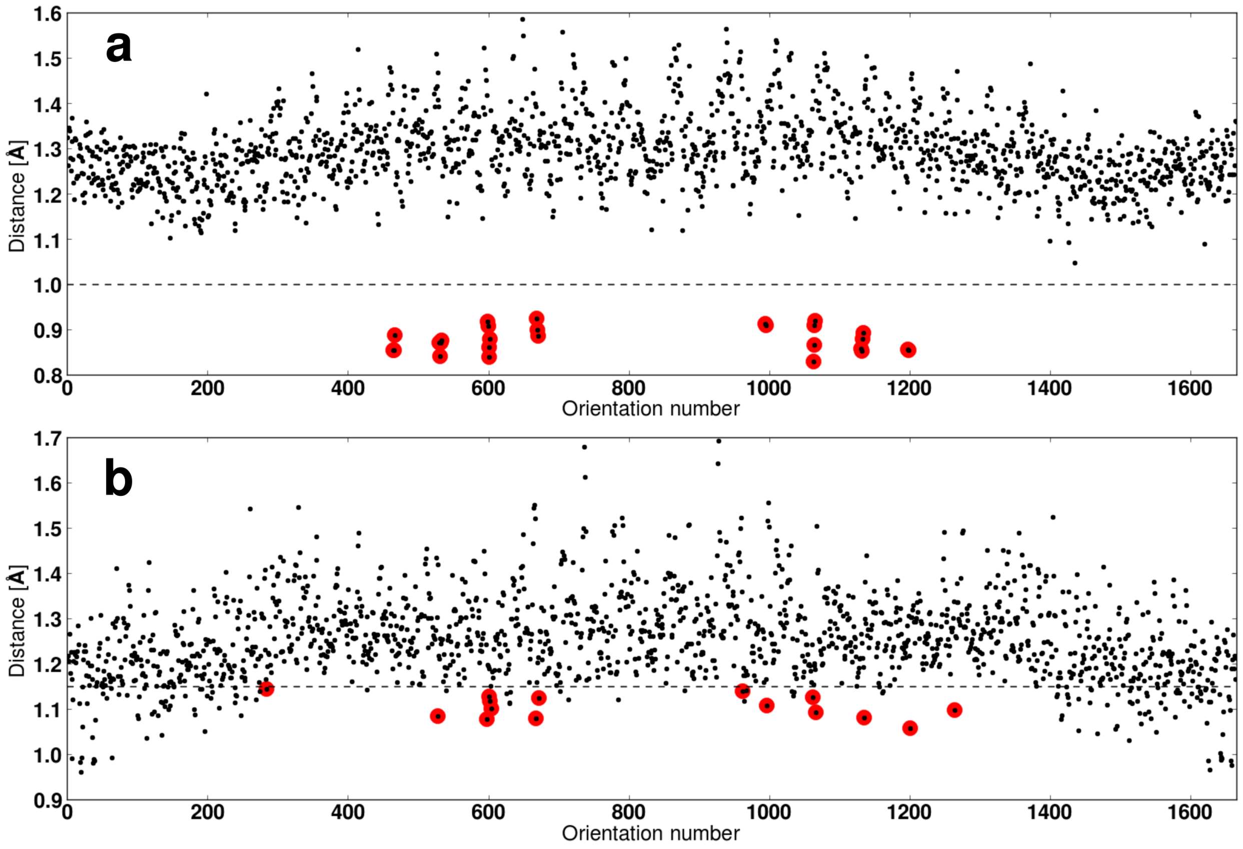

Supplementary Figure 8. A test for the facial recognition algorithm. (**a**) The reference set of coordinates was obtained by determining 10 maxima and 3 minima from the calculated STM image number 600, each of which were then randomly deviated in the range [0, 1] Å to a random direction. This set of points was compared to each calculated STM image. (**b**) Similar to (**a**) with the deviation range of [0, 2] Å. The red data points correspond to a perspective very close to the reference ie. orientation number 600.

a **b** **c**

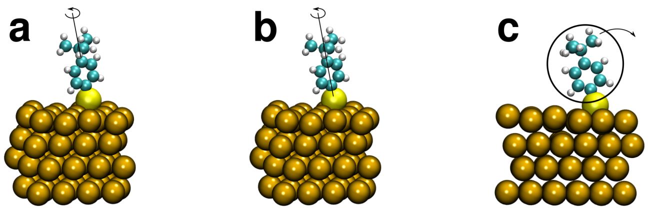

d

	E_a (eV)	L-He	L-N	300 K
a	0.08	10^{-102}	10^{-6}	10^{-2}
b	0.04	10^{-51}	10^{-3}	10^{-1}
c	0.27	10^{-345}	10^{-18}	10^{-5}

e

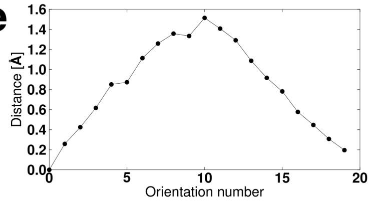

f

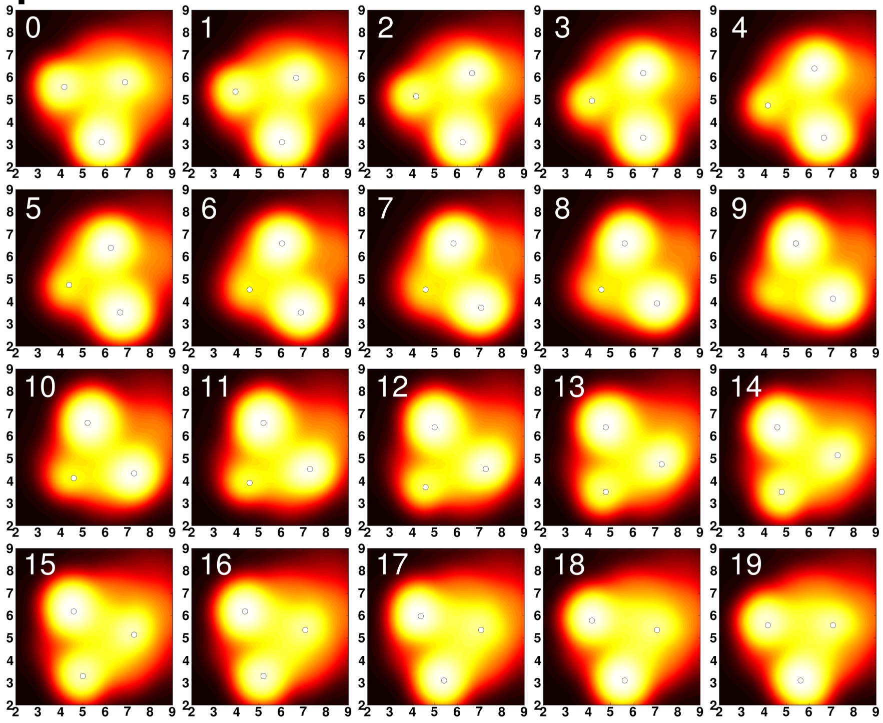

Supplementary Figure 9. Determining the rotational barriers and rotation probabilities of a single TBBT molecule absorbed onto bridge site of Au111 surface. (**a**,**b**,**c**) Illustrations of the different rotations that were considered: (**a**) Rotation of the tert-butyl head group. (**b**) Rotation of the thiol around the principal axis. (**c**) Rotation of the thiol around the axis parallel to the surface. (**d**) Rotational barriers and temperature-dependent Boltzmann factors in the Arrhenius equation for rotations a, b and c, referring to the corresponding images. The factors are shown for temperatures of liquid Helium, liquid Nitrogen and 300 K. (**e**) Results of the extremum coordinate comparison for 20 rotations of type **a** where the coordinates of the three maxima, shown for each state in the calculated STM images (**f**), are compared to the initial state (state number 0) without minimizing with respect to rotations. The maximum distance in (**e**) is 1.5 Å and the mean value 0.8 Å. X-Y scales in (**f**) are shown in Å.
